# *In vitro* assessment of surface roughness and microbial colonization on nickel titanium wires obtained from different manufacturers

**DOI:** 10.6026/973206300212510

**Published:** 2025-08-31

**Authors:** Kinnari Shah, Mahin Shaikh, Sejal Singh, Janvi Meena, Radhika R. Agnihotri, Rutu Paresh Shah

**Affiliations:** 1 Department of Orthodontics & Dentofacial Orthopaedics, Karnavati School of Dentistry, Gandhinagar, Gujarat, India-382422; 2 Private Practitioner, Ahmedabad, Gujarat, India; 3 Department of Prosthodontics, AMC Dental College and Hospital, Ahmedabad, Gujarat, India

**Keywords:** Surface roughness, surface free energy, orthodontic archwires, nickel titanium, bacterial adhesion

## Abstract

Nickel-titanium wires and Copper nickel-titanium wires obtained from three different manufacturers. They were divided into three groups: Group 1 - GAC (Sentalloy®), Group 2 - ORMCO^TM^, and Group 3 - American Orthodontics (AO)®. Further, they were tested for surface roughness using a Profile projector. *Streptococcus mutans* adhesion on wires was tested by inoculating the wires in a saliva sample after isolating the bacteria by overnight incubation of the sample in blood agar. Surface roughness and bacterial adhesion were significantly greater in copper nickel titanium wires of all the three companies; rectangular wires show greater surface roughness and adhesion than round wires. Thus, increase surface roughness of orthodontic wires lead to increased bacterial colonization, affecting their biocompatibility.

## Background:

Orthodontic appliances serve as retentive areas for plaque accumulation, promoting increased adhesion and colonization of microbes. *Streptococcus mutans* (*S. mutans*) is abundantly present in plaque in patients with orthodontic appliances [1, 2, 3, 4, 5, 6- 7]. Different components of fixed orthodontic appliances, such as archwires, brackets, and ligation methods, significantly impact bacterial adhesion and plaque buildup, which increases the risk of white spot lesions, gingival inflammation and dental caries during orthodontic treatment [8, 9- 10]. In fixed orthodontic treatment, three types of wire materials are commonly used: stainless steel, β-titanium, and nickel-titanium (NiTi) alloy. β-titanium wires are generally used briefly during the final treatment phase, whereas NiTi and stainless steel wires play a more prolonged role. NiTi wires are primarily used in the NiTi alignment stage, while stainless steel wires are the main working wires in later stages [11]. Since these wires remain in the oral cavity for an extended period and directly impact treatment efficiency, their mechanical properties are crucial. Rougher surfaces of the orthodontic wires increase surface free energy and bacterial adhesion, preventing the bacterial colonies from dislodgement [4, 5, 6, 7, 8, 9, 10, 11-12]. The biocompatibility of orthodontic archwires depends on their surface roughness, which influences other properties such as friction, spring back, esthetics, *etc.* [13, 14, 15, 16-17]. Additionally, the biofilm formed by *S. mutans* causes corrosion of the appliances used [18, 19]. In fixed appliance therapy using sliding mechanics, the primary resistance to tooth movement comes from the friction between the bracket slot and the archwire. To ensure effective tooth movement with an optimal biological response, friction should be minimized, allowing for the application of lower force levels. The surface characteristics of orthodontic archwires, such as roughness, hardness and topography, affect sliding mechanics by influencing friction levels. Additionally, these properties impact the aesthetic quality, corrosion resistance, and biocompatibility. Various techniques, including laser spectroscopy, contact-surface profilometry, and atomic force microscopy (AFM), are used to assess the surface roughness of orthodontic archwires [20, 21]. A study by Bourauel *et al.* compared these methods and found that their results were generally consistent [22]. Therefore, it is of interest to investigate the difference in the surface roughness of nickel-titanium (NiTi) archwires of different crystallographic forms obtained from different manufacturers and the respective adhesion of *S. mutans*.

## Material and Methods:

Nickel-titanium wires - 0.016 inch NiTi (n=10), 0.019 x 0.025 inch NiTi (n=10) and 0.019x0.025 inch Copper (Cu)NiTi (n=10)- obtained from three different manufacturers were divided into three groups: Group 1 - GAC (Sentalloy®), Group 2 - ORMCO^TM^, and Group 3 - American Orthodontics(AO)®.

## Surface roughness test:

The as-received wires were segmented into approximately 10 millimeters, and their surfaces were examined at each millimeter using a Nikon V-12 Profile Projector (Figure 1 - see PDF).

## *Streptococcus mutans* adhesion test:

The saliva sample for the bacterial adhesion test was obtained from a healthy adult patient with clinically healthy periodontal status, no craniofacial malformations or abnormalities, and no ongoing treatment for conditions that may affect salivary enzymatic activity. The patient provided written informed consent before the sample collection. The saliva sample was inoculated in blood agar to isolate the streptococcal strains. The suspension of streptococcus was made by re-inoculating the isolated bacterial species in serum broth. Each wire sample was divided into a 10-mm segment and the specimens were immersed in vials, each containing 1 mL of bacterial suspension. Vials contained the bacterial suspension along with the wire specimen. The control group comprised 1 mL of bacterial suspension without a wire specimen (Figure 2a - see PDF). All vials were incubated overnight at 37°C (Figure 2b - see PDF). After overnight incubation, subculturing was performed in Blood agar from all vials and incubated overnight at 37°C to measure the colony-forming units of streptococcal mutans from each vial. The number of bacteria expressed as colony-forming units (CFU)/milliliter (mL) was counted for each vial and compared with the bacterial suspension in the control vial.

## Statistical analysis:

The surface roughness and Streptococcus adhesion test data were analyzed using an independent sample T-test. A one-way ANOVA (Analysis of variance) test followed by post-hoc Tukey test was used to analyze the differences in surface roughness and bacterial adhesion between the groups. Statistical analysis was conducted using the Statistical Package for Social Sciences (SPSS) for Windows v.22.0 (SPSS Inc., Chicago, IL, USA). A p-value of <0.05 was considered as statistically significant.

## Results:

The surface roughness of wires in Groups 1, 2, and 3 can be seen in Figures 3-5 (see PDF), respectively. Table 1 (see PDF) shows the independent T-test results between the surface roughness of 0.016 inch NiTi and 0.019x0.025 inch NiTi arch-wires of all groups. 0.016 inch arch-wires show less surface roughness when compared to conventional rectangular wires, with Group 3 demonstrating a statistically significant difference (p<0.0001). Groups 2 and 3 show a significant difference in the surface roughness between 0.019x0.025 inch conventional and Copper NiTi wires with a p-value of 0.05 and <0.0001, respectively (Table 2 - see PDF). The bacterial adhesion in the control group can be seen in Figure 6 (see PDF). Figures 7-9 (see PDF) show the adhesion on wires of all three groups. Tables 3 (see PDF) and 4 (see PDF) show the independent T-test results of bacterial adhesion between the groups. Rectangular arch-wires show statistically significant bacterial adhesion compared to round wires, with maximum adhesion on Copper NiTi wires (p<0.0001). Table 5 (see PDF) shows the result of a one-way analysis of variance (ANOVA) for comparing the surface roughness between the groups for all three arch-wires, followed by a Tukey HSD post hoc test (Table 6 - see PDF) to evaluate pairwise differences among the groups. Tukey HSD post hoc test demonstrated significant differences in the surface roughness of 0.016 inch NiTi wires of Groups 1 and 2 when compared to Group 3 (p < 0.001). ANOVA test for 0.019 x 0.025 inch NiTi wires showed significant variation between the groups, with post hoc test suggesting the difference to be statistically significant between groups 2-3 and groups 1-3 (p<0.0001). Post hoc Tukey test for 0.019 X 0.025 inch Copper NiTi wires showed significant difference only between Groups 2 and 3 with a p-value of 0.004. A one-way analysis of variance (ANOVA) test compared the microbial colonization among the three experimental groups and the control (Table 7 - see PDF). Post hoc comparisons using the Tukey HSD tests are summarized in Tables 8 (see PDF) and 9 (see PDF). The Post hoc test for 0.016 inch NiTi revealed a statistically significant difference among the groups (p < 0.0001), with Group 1 exhibiting significantly higher mean values (p < 0.001). The Control group also demonstrated significant differences when compared to Group 2 (p < 0.001) and Group 3 (p < 0.01). Additionally, Group 2 showed a significantly higher mean than Group 3 (p < 0.05). Post hoc Tukey HSD test for 0.019x0.025 inch NiTi confirmed that all pairwise comparisons between groups were statistically significant (p < 0.0001); with the highest mean difference observed between Group 2 and the Control group. Post hoc analysis for 0.019 x 0.025 inch Copper NiTi wires suggested that the Control group exhibited a significantly lower mean when compared to all experimental groups (p < 0.001), indicating higher *S. mutans* adhesion on Copper NiTi wires of all groups, co-relating with the increased surface roughness of Copper NiTi wires.

## Discussion:

The archwires used in the iNiTial stages of orthodontic treatment should have low stiffness to generate light force while also possessing high strength to prevent permanent deformation when engaged in severely crowded teeth. This study aimed to assess the surface roughness and *Streptococcus mutans* adhesion on archwires from different manufacturers to examine whether increased surface roughness promotes increased bacterial colonization. For this study, three types of wires - 0.016 inch NiTi, 0.019 x 0.025 inch conventional NiTi, and 0.019 x 0.025 inch Copper NiTi- were examined after being procured from three manufacturers, namely, GAC (Sentalloy®), ORMCO^TM^, and American Orthodontics(AO)®. The results showed that the 0.016 NiTi wire from American Orthodontics exhibited lower surface roughness compared to the 0.019 x 0.025 inch conventional NiTi. The profilometry test for assessing surface roughness requires a flat surface; however, the round archwires exhibit a curvature on their surface, resulting in a lower roughness measurement when compared to rectangular wires. Therefore, careful evaluation of their surface roughness is necessary. Sarul *et al.* analyzed surface roughness through texture and fractal analysis, revealing considerable variations in the surface characteristics of orthodontic wires. They concluded that even wires of the same type from the same manufacturer can have significantly different surface roughness [23]. When comparing 0.019 x 0.025 inch Copper NiTi wires with 0.019 x 0.025 inch conventional NiTi, Copper NiTi exhibited greater surface roughness across all three groups. These findings are consistent with a study by Gravina *et al.* that reported an increase in the surface roughness of Copper NiTi wires. They also observed that Cu-NiTi (35°C) archwires showed uneven surface morphology, characterized by features such as grooves, indentations and micro cavities formed due to the pullout of particles [24]. In their study to evaluate surface roughness using an Atomic Force Microscope (AFM), Mohan *et al.* found that HANT wires had greater roughness compared to conventional NiTi archwires [25]. Due to the reactivity of Copper, the dangers of surface cracking, porosity, and the formation of internal cavities are high. This may account for the increase in surface roughness observed in CuNiTi wires. Bourauel *et al.* examined the surface roughness of various orthodontic archwires and concluded that NiTi wires exhibited a wide range of surface roughness across different manufacturers, likely due to variations in manufacturing techniques and final polishing processes [22]. These results align with the findings of our study, which also demonstrate variations in surface roughness among wires of similar cross-sections from different manufacturers. The other objective of the study was to determine the difference in bacterial adhesion on the wires in the three groups. Bacterial adhesion was significantly higher in Copper NiTi compared to conventional.

NiTi archwires amongst the round and rectangular NiTi, bacterial adhesion was more pronounced in rectangular archwires. These findings are similar to a study carried out by Abraham *et al.* wherein they compared the bacterial adhesion on Copper NiTi and conventional NiTi of round and rectangular cross-sections of ORMCO^TM^ company and concluded that Copper NiTi wires show more bacterial adhesion compared to conventional NiTi, and the difference was statistically significant for both the cross sections studied [26]. Comparing round and rectangular archwires for bacterial adhesion, rectangular conventional and Cu NiTi showed significantly increased adhesion. Quirynen *et al.* concluded that surface roughness plays a significant role, since a decrease in surface energy and surface roughness reduces plaque formation [27]. This finding confirms the results of the present study. Orthodontic archwires play a significant role in plaque accumulation; therefore, one should be aware of bacterial adhesion to select an archwire type that attracts less biofilm and has appropriate antibacterial properties. Higher levels of bacteria increase the acidity of dental plaque in orthodontic patients due to lactic acid formation that can lead to the appearance of white spot lesions within one month of bracket placement [28, 29-30].Coated NiTi wires exhibit enhanced antibacterial anti biofilm properties with inhibition against Gram-positive and Gram-negative bacteria [31]. Osmani *et al.* concluded in their study that a change in salivary pH does not induce a significant change in the mechanical properties of the uncoated NiTi; however, it does affect the coated wires [32]. *In vivo* examination of esthetic-coated archwires showed a similar risk of microbial adhesion as non-coated wires [33, 34, 35, 36-37]. However, certain studies have demonstrated an increase in bacterial accumulation on uncoated wires [38, 39]. In this study, NiTi wires of American Orthodontics (AO) ®, demonstrated comparatively less surface roughness and bacterial adhesion. In a study by Sahu *et al.* nickel-titanium and beta-titanium wires exhibited increased roughness and higher friction levels after intraoral use [40]. Hence, we can conclude that clinical observation of orthodontic archwires over a long period is necessary to obtain accurate information on the surface characteristics of materials and their susceptibility to bacterial adhesion.

## Limitations of the study:

This study is an in-vitro study and does not take into account the various intraoral conditions, such as viscosity of saliva, type of food intake, oral hygiene, any hormonal disorder which may affect the saliva characteristics, lead to changes in the surface-free energy and roughness and alter the amount of adhesion of bacteria.

## Conclusion:

Surface roughness was higher in the Copper NiTi wires of all three companies. The adhesion of *S. mutans* was increased in Copper NiTi wires of all companies when compared to 0.016 NiTi and 0.019x0.025 NiTi. Among round and rectangular wires, rectangular wires showed greater surface roughness and bacterial adhesion due to increased surface area. Thus, Copper NiTi wires have greater surface roughness, which may have contributed to an increased adhesion of *Streptococcus mutans*.
